# Mother Like Mothers and Work Like Fathers: U.S. Heterosexual College Students’ Assumptions About Who Should Meet Childcare and Housework Demands

**DOI:** 10.1007/s11199-021-01252-3

**Published:** 2021-10-28

**Authors:** Annie McConnon, Allegra J. Midgette, Clare Conry-Murray

**Affiliations:** 1 Department of Psychology, Saint Joseph’s University, Philadelphia, PA USA; 2 Department of Psychological and Brain Sciences, Texas A&M University, College Station, TX USA; 3 University of North Carolina at Chapel Hill, Frank Porter Graham Child Development Institute, Chapel Hill, NC USA

**Keywords:** Work-life balance, Gender roles, Division of household labor, Childcare, Gender, Family, Marriage, Attitudes, Thematic analysis

## Abstract

Many U.S. women report balancing competing demands for labor within the family and the workplace. Prior research has found that young adult heterosexual U.S. women are still anticipating doing the majority of their future family’s childcare and housework, though they hold more progressive gender role attitudes than in the past. The aim of the present study was to investigate the assumptions of 176 heterosexual college students in the U.S. (*M* age = 20.57, 88.64% European American, 51.70% ciswomen, 48.30% cismen) about how childcare and housework should be balanced in the context of work responsibilities. Participants were asked to rate their level of agreement with two items about working mothers and childcare and working fathers and household care, and provided open-ended responses to explain their justifications for their rating. Open-ended responses were thematically coded. Results revealed that most participants wanted mothers to have the choice to work but considered childcare a limiting problem that (primarily) mothers should solve. Similarly, participants believed that working full-time did not excuse a husband from helping with chores, however they did not express concerns with the term “helping” which implies that the husband would not hold any primary responsibility. Overall, the findings suggest the importance for educational and policymaking interventions and future research to highlight practices that support and encourage the role of men in addressing childcare and household needs.

Many women report having to balance competing demands for care labor within the family and paid labor within the workplace (Perry-Jenkins et al., 2007; Shockley et al., 2021). This dual demand for labor has direct consequences for women’s involvement in the labor force (Christnacht & Sullivan, 2020), women’s adjustment to becoming new parents (Perry‐Jenkins et al., 2007), women’s rates of depression (Perry‐Jenkins et al., 2007), marital satisfaction (Li et al., 2020), women’s time poverty (Hyde et al., 2020), and women’s career aspirations (Drinkwater et al., 2008). For instance, married women in the United States in heterosexual relationships who lost access to childcare as a result of the COVID-19 pandemic were more likely to take on childcare duties while also working remotely, and this dual labor was associated with a negative impact on women’s well-being as well as their career performance (Collins et al., 2020; Shockley et al., 2021). Moreover, heterosexual emerging adult women, many of whom grew up observing their mothers taking on this double burden (Drinkwater et al., 2008), also report expecting an unequal and gendered division in their future households (Askari et al., 2010; Dernberger & Pepin, 2020; Fetterolf & Eagly, 2011). However, recent research has found that this expectation occurs despite emerging adults’ holding egalitarian attitudes (Croft et al., 2020).

The gendering of labor in the context of balancing work and family life demands has implications not only for women’s well-being, but also the structuring of class, gender, and race relations within the U.S. (Coltrane, 2000; Glenn, 2010). The family has been considered one of the main sites of women’s oppression, as it is the “primary arena where men exercise their patriarchal power over women’s labor” (Hartmann, 1981, p. 377). In the United States, and abroad, women in heterosexual relationships continue to take on most of their household’s labor (Jansen et al., 2016; Lachance-Grzela & Bouchard, 2010; Mandel & Lazarus, 2021; Organization for Economic Co-operation and Development [OECD], 2019). The gendered division of labor has been linked to women’s rates of depression, marital conflict, as well as supporting gender inequalities in labor outcomes (Coltrane, 2000; Ferrant et al., 2014). In the U.S., women spend an additional two 40-h weeks per year of labor compared to men, when the average time each gender spends on both paid and unpaid labor (home and workplace) is considered (OECD, 2019). Overall, prior research suggests that women’s continued greater involvement in domestic labor is one of the main indicators of the “stalling of the (gender) revolution” (England, 2010; Hochschild & Machung, 2012).

In the current study, we use thematic analysis to examine U.S. heterosexual college-attending emerging adults’ assumptions about how the demands for home labor (i.e., childcare and housework) should be balanced in the context of women’s and men’s involvement with the workplace to gain better insight into why emerging adults may anticipate and reproduce gender unequal practices in their future households. Prior research with college-attending young adults found that, particularly heterosexual women, still anticipate serving as the primary caregiver in their future families (Croft et al., 2020) or performing the majority of their future family’s childcare and housework (Askari et al., 2010; Fetterolf & Eagly, 2011), and these expectations are proving to be difficult to change (McLean et al., 2017). Investigating emerging adults as a population (18–25 years old) is particularly important because they are in a period of transition and how they understand and make sense of how paid work and care work should be balanced is likely to affect their division of labor in their future homes (Askari et al., 2010).Therefore, investigating gender ideologies in the form of open-ended questions (Davis & Greenstein, 2009) can provide much needed insight into how emerging adults make sense of gender roles, the gendering of labor, and balancing work and life demands.

Moreover, though women across racial-ethnic groups (Perry-Jenkins et al., 2013; Wight et al., 2013) and social class (Miller & Carlson, 2016) tend to take on more of the childcare and household labor in heterosexual relationships (Bianchi et al., 2012; Goldberg et al., 2012), White middle-class women with higher levels of education are more likely to buy themselves out of doing household labor and hire working-class women of color to provide the services (Glenn, 1992, 2010). Data collected in the United States from 2010–2019 shows that primarily White, wealthy cities employ the highest number of nannies and house cleaners (Economic Policy Institute [EPI], 2020). Nonetheless, on average White women contribute to domestic labor about 1.6 times more than White men (Bianchi et al., 2012; Wight et al., 2013). At the same time, college-educated men have been found to be more willing to help with domestic labor when compared to non-educated college men (Miller & Carlson, 2016). Furthermore, in addition to survey data, scholars have argued that it is “crucial that we refine our understanding of the underlying *meanings and purposes* given to household labor that help perpetuate men’s and women’s gendered allocation” (Lachance-Grzela & Bouchard, 2010, p. 777). Therefore, thematic analysis of the reasoning of students attending a Primarily White Institution (PWI), may also aid understanding of why well-educated and White middle-class heterosexual families may outsource and contribute to the gendered, classed, and raced nature of labor.

## Many Women are Still Doing the Double-Shift

Women are becoming more and more involved in the workforce over time. U.S. women’s overall labor force participation across racial-ethnic groups between the ages of 24–54, was about 15% lower than men in 1999, about 13% lower than men in 2019, and anticipated to be about 11% lower than men in 2029 (U.S. Bureau of Labor Statistics, 2020). While American women’s labor force participation rates are increasing, their experiences in the labor force are still gendered, and as a result, women face the double burden of both paid and unpaid labor. In fact, one third of working women in 2018 in the U.S. were mothers (Christnacht & Sullivan, 2020). Working mothers with children under the age of six have reported that they reduce work hours to part-time or discontinue working altogether due to childcare responsibilities (Christnacht & Sullivan, 2020). Moreover, according to a study of 21 American heterosexual couples (other demographics not reported) where the woman was earning or had earned 80–100% of the family’s income, 43% of women reported that they felt added pressure and demands such as housework and childcare in addition to the primary breadwinner role (Chesley, 2017). This gender inequality in the home leads to work-life inequalities for U.S. women, as they not only contribute about 96 min more unpaid labor per day than men, but also spend about 49 min less than men on leisure activities (i.e., hobbies, watching tv, outdoor activities; OECD, 2019). Overall, prior research suggests that women are often engaged in doing the double shift of paid and unpaid labor.

## Expecting an Egalitarian Workplace and a Traditional Family Life

Gender role attitudes regarding engagement in the workplace and family life have also changed. According to a gender ideology survey of American 12^th^ graders in public and private schools from 1976–2014 (42% White men, 43% White women, 6% Black men, 8% Black women), earlier generations expected women to be primarily “homemakers,” and considered a traditional arrangement of the husband working outside the home and the wife working inside the home as most desirable (Dernberger & Pepin, 2020). However, emerging adults’ support of women’s involvement in the workplace (e.g., husband and wife should both work outside the home) increased over time, as 8% of the sample found this acceptable in 1976 compared to 24% in 2014 (Dernberger & Pepin, 2020). Additionally, a separate analysis of surveyed American 12^th^ graders from 1976–2015 (42% White men, 44% White women, 6% Black men, 8% Black women) found that whereas support of gender equality in the workplace was relatively high and increased over time, egalitarianism within the home (sharing of household duties) did not receive similar levels of increased support over time (Pepin & Cotter, 2018). Therefore, the increased support for equality in the workplace did not reflect similar levels of support for equality within the home.

Similarly, European- and Asian-Canadian college students have been found to be, on average, supportive of egalitarianism in the home and in the workplace (Gere & Helwig, 2012). However, analysis of students’ underlying reasoning showed that when presented with traditional gender roles (e.g., the wife should be primary caregiver), participants often referred to traditional gender role stereotypes and expectations (e.g., women are better at caring for children). Thus, although emerging adults may seem to be egalitarian in their attitudes, initial investigation into their underlying reasoning suggests that they are still influenced by and rely on traditional gender role stereotypes to make sense of how paid and unpaid labor should be divided. However, the study did not investigate in-depth emerging adults’ assumptions of how couples should balance both work and family life demands.

A recent thematic analysis of emerging adult expectations for their future found differences in how emerging adults approach balancing career and family life. Whereas some were certain that it is possible to “have it all,” others were not so sure (Ezzedeen et al., 2018, p. 570). Ezzedeen et al.’s (2018) investigation into Canadian college women’s (*M*_age_ = 21; 35.8% South Asian, 23.8% White, 13.4% Asians, 10.4% Middle Easterners, 10.4% West Indians, 3% Africans, and 3% Latinx) expectations for their future plans and expected ability to both work and have children, found that whereas some women believed they can “have it all” some referenced the notion that sacrifices would have to be made in order to balance work and career given that parenting, too, is a full-time job. Some noted that having children limited career growth due to maternity leave and the responsibility of caretaking. Similarly, Coyle et al. (2015) found that although most college-attending U.S. emerging adults (*M*_age_ = 19.8, no racial-ethnic demographics reported) planned to work, women were more likely to plan to alter their work and family life in response to expectations for work-family conflict, and to plan to work part-time rather than full-time before their children were preschool aged. Moreover, those who expected to work part-time or full-time expected more work-family conflict than women who planned to not work while their children were young. Of relevance to the current study, women were more likely to report anticipating that family life would impact work life, whereas men anticipated work life would impact family life.

Overall, the research suggests that emerging adult women expect to take on most of the childcare and housework (Askari et al., 2010; Erchull et al., 2010; Fetterolf & Eagly, 2011). At the same time, emerging adult women also desire to work (Fulcher et al., 2015), understand that work is good for their well-being (Fetterolf & Eagly, 2011), and believe that a work-life balance is achievable (Ezzedeen et al., 2018; Gerson, 2010). Because emerging adult women are less likely to endorse traditional gender role attitudes, but are expecting inequality in their future, more gender egalitarian attitudes are not necessarily predictive of an expected future egalitarian division. Prior quantitative and qualitative research suggests that emerging adults recognize a tension between maintaining and caring for the family and participation in the labor force. Less is known about the underlying assumptions that may be informing the decisions by emerging adults about how heterosexual couples *should* address the conflict between the dual demands of work and family.

## The Present Study

To better understand how emerging adults’ make sense of how conflict between family and work life should be addressed, the present study addressed two main questions. What are participants’ implicit and explicit assumptions regarding how: 1) childcare should be balanced in the context of women’s involvement in the workplace? and 2) how the need for housework should be balanced in the context of men’s involvement in the workplace? Answering these questions also allows for an investigation of assumptions about what aspects of the workplace influence family involvement and what aspects of family life influence workplace involvement and the potential role of a partner in helping navigate these dual demands.

## Method

The data analyzed in the present study is part of a larger dataset (see Midgette & D’Andrea, 2021) that set out to investigate emerging adults’ expectations for and sense-making about the gendered division of household labor.

### Participants and Recruitment

A total of 176 emerging adults participated in this study. Participants had a mean age of 20.57 years (*SD* = 1.18, *range* = 18, 23). This study was a secondary data analysis of open-ended responses from a larger quantitative-centered program of research; thus the stopping point of data collection was determined by a power analysis. As a result, we did not aim for data saturation (Braun & Clarke, 2021). A little over half of participants (51.70%) identified as ciswomen, and the remainder identified as cismen. Most participants (88.64%) identified as European American, 3.98% identified as Asian American, 3.98% identified as Latinx, 2.84% identified as African American, and .57% preferred not to say. All participants identified as heterosexual. Most participants (90.34%) said they expected to be married in the future, whereas 9.66% stated they might be married in the future. Moreover, 48.86% reported having a mother who worked full-time when they were growing up, whereas 23.30% had a mother who worked part-time, 27.27% had a stay-at-home mother, and .57% (*n* = 1) did not live with their mother while growing up. Many of our participants (81.25%) came from a household with married parents. A small portion of the participants (15.9%) came from a household with divorced parents, 1.14% with a widowed parent, .57% with a single parent, and 1.14% with remarried parents.

### Procedure

During the Spring of 2020, participants attending a PWI in Philadelphia, U.S. were invited either through email or as part of the psychology department’s subject pool to participate in this study. Participants filled out a series of measures through a Qualtrics survey, one of which included a measure of their gender ideology attitudes and justifications (described in more detail below). This study titled, “Reasoning and Life” (IRB Protocol # 1510757), received IRB approval from Saint Joseph’s University prior to data collection. Following Gere and Helwig’s (2012) methodology, participants were presented with several items related to gender roles within the family, some of which were egalitarian and others traditional, and asked to rate their agreement with each item on a scale from 1 (*strongly disagree*) to 7 (*strongly agree*), and then to justify their rating, “Please explain why you agree/disagree with the above statement as clearly as you can.” For the purposes of the present study, we analyzed the justifications given for the following two items: “Married women who have preschool-aged children should not work outside the home” (Hardesty & Bokemeier, 1989) and “A wife should not expect her husband to help around the house after he comes home after a hard day’s work*”* (Cunningham, 2008). These items were chosen because they indicate the degree to which women and men in a heterosexual marriage should take on the responsibility for childcare and housework in their roles as spouses and parents. To use shorthand, we call these the Working Mom Beliefs and Husband Does Housework Beliefs, respectively. The wording of the items was also intentional. For instance, prior research has shown that the word “help” is often used by participants to describe men’s lower involvement in household labor and as a means for accepting and perpetuating a gendered division in the home (Midgette, 2020; Miller & Carlson, 2016). Participants’ open-ended responses to both items were coded thematically. All but six participants provided an open-ended response for the childcare item, and all but two participants completed the open-ended justification for the housework item.

### Thematic Analysis

In order to investigate the content of emerging adults’ justifications, we employed a thematic analytical approach to systematically investigate patterns of shared meanings in the data (Braun & Clarke, 2006). All three authors identify as cisgender women. The first author identifies as White and is a first-year graduate student who was an undergraduate at the time of data analysis and writing. The second author is a multiracial Brazilian American Visiting Assistant Professor in Psychological and Brain Sciences and married to a man. Her area of study is focused on how individuals across cultures make sense of the gendering of labor within heterosexual households. The third author identifies as White and is an associate professor of psychology who studies gender and moral development and is married to a man. In order to address biases and differences in interpretation we had discussions about the analyses and framing of the results, with the goal of presenting our shared interpretation of what participants presented in their responses.

Following procedures described by Boyatzis (1998), a team comprised of the first two authors and another coder collaboratively developed two codebooks based on reading the two types of open-ended responses (childcare item and housework item). Each codebook included code names, definitions, and examples taken from the data. Codes were created and applied to the open-ended responses and modified or added following the reading of new responses. After the codebooks were developed based on the reading and application of codes to all responses, two coders independently read and coded 20% of the responses for each item to establish reliability. Final coder agreement, as calculated through Cohen’s Kappa, for the childcare and housework item responses were κ = .81 and κ = .84, respectively. All disagreement was discussed and resolved following consensus. Once reliability was established, the remainder of the responses were coded by the two coders. Our analysis focused on the two guiding research questions. Following the application of codes, themes were created based on code frequency, shared meaning, and the co-occurrence of codes. The most frequent and infrequent codes were counted and tabled to know which ideas were present throughout responses and which ideas were rarer. To create themes, original text responses were collated next to their coding category, which allowed for investigating when textual responses had co-occurring codes within and across coding categories. Text and codes were analyzed for patterns of shared meaning and underlying assumptions in relation to our research questions. The examples in the results section use the participants’ own words and occasionally include their errors and typos. We identified the gender of the participant (W = woman; M = man) for all material presented in the results.

## Results

### Analysis of the Working Mom Beliefs

Many participants disagreed with the idea that married women who have preschool-aged children should not work outside the home. Overall, 73.30% participants (52.7% women) either strongly, moderately, or slightly disagreed, whereas 8.52% (46.6% women) neither agreed nor disagreed, and the remaining 18.18% (50% women) either somewhat, moderately, or strongly agreed. Therefore, most participants believed that married women with preschool-aged children should be able to work outside the home. Analyses revealed five major themes associated with participants’ assumptions underlying their views and explanations for their position: 1) work as a personal (protected) choice, 2) mothers should work as working fathers do, 3) where are the fathers? childcare is mom’s (not dad’s) problem, 4) mothers can’t choose to stay home, and 5) mothers should stay home. Each theme is considered in more detail below. (See Table 1 for a summary of major themes and code frequency).

**Table 1:** Themes and Coding Analysis for Working Mom Beliefs

**Major Themes**				
*Codes*	Description	Cohen’s Kappa	Counts/ Percentages	Exemplary Quote
**Work as a Personal (Protected) Choice**				
*Women can do what they want*	Refers to women as the ones that should decide if they want to work	.74	*33 (18.75%)*	“I disagree with the statement because women should be allowed to do as they wish.” M
*Women should not be required to stay in the home*	Refers to the idea that women should not be required or restricted to staying home and being the primary caretaker of children	.85	*38 (21.6%)*	“No matter how old your children are the women should still be able to work. They should not have to stay in the home.” M
**Mothers Should Work as Working Fathers Do**				
*Wonder woman*	Refers to the ability of women to both work while having children and be successful in doing both	.82	*22 (12.5%)*	“I think that married women with small children are able to both work and be a mom successfully.” W
*If men can do it, women can too*	Refers to the idea that women can or should be held to the same standard as men	1.00	*12 (6.8%)*	“I disagree because I think that women should be able to chase their career just the same as men. I think that if they coordinate timing and picking the child up there shouldn't be an issue. I think that they can also get a nanny or alternate days of being at home.” W
**Where are the Fathers? Childcare is Mom’s (Not Dad’s) Problem**				
*There are systems in place that allow women to work*	Refers to systems outside the immediate family (nanny, daycare, school, etc.) that allow women to work	.58	*27 (15.3%)*	“Just because a mother has small children, does not mean she should be restricted to working at home. The child can go to preschool, have an after school babysitter, or their father to help watch them, not just the mother. She should be allowed to choose the job she finds to be best for her and her family.” W
*Children should not be a limitation*	Refers to the idea that children don’t or should not influence or limit women’s ability to work	.87	*24 (13.6%)*	“Having young children does not mean you cannot work outside the home or still be focused on your career.” W
*Childcare is a shared responsibility*	Refers to men and women as responsible for sharing the responsibilities of childcare	1.00	*4 (2.3%)*	“I believe that they should be allowed to work outside the home because it is only fair given that nobody ever questions men with preschool children working outside the home. Spouses should share the caregiving, neither should have to sacrifice their career.” W
**Mothers Can’t Choose to Stay Home**				
*Staying home is not always an option*	Refers to limitations that do not allow a mother to have the choice to stay home as the primary caretaker	.65	*15 (8.5%)*	“I think the mother should have a choice if they would like to work or not. Sometimes, a mother might need to work if they are a single mother.” M
*Breadwinner*	Refers to the idea that women both can and should financially provide for the family	1.00	*20 (11.4%)*	“I feel that even though a mother has a child who is young, the pressure should not solely rely on the father for earning money, especially if the mother enjoys working. It should be decided between the parents.” W
**Mothers Should Stay Home**				
*Well -being of child*	Refers to the well-being of the child as a reason for why women should do childcare and/or be the primary caretaker	1.00	*14 (8.0%)*	“Mostly this is because I was raised this way. If both spouses are financially secure enough to support one spouse staying home, I believe the mother should do it. Especially at such a young age, those moments are important for a child's growth.” M
*Gender Roles*	Refers to the traditional role of the woman as the primary caretaker of children	1.00	*25 (14.2%)*	“B/c women are the primary caretakers, also need to breastfeed so I think that's important.” M

### Work as a Personal (Protected) Choice

Reflecting the position that mothers should be able to work was the assumption that women should be able to choose to do whatever they wanted. Participants agreed that whether a woman works is her own personal choice and should not be up to anyone but herself. For example, one emerging adult man (M) stated, “it is not my decision to decide if a woman wants to work or not.” Echoing the assumption that working is the choice of the woman, an emerging adult woman explained:

I disagree that women shouldn't work outside the home with preschool aged children because I think it is a choice someone makes on their own. I do not think anyone should feel like they cannot work outside the home because some families have to have both parents working to make enough to support the family. (W)

The fact that work should be a woman’s choice was also often mentioned in response to the assumption that women might be obligated to go against their wishes. In particular, the belief that women should not feel pressured to stay home was common among women participants, along with the belief that women should not be expected or required to stay in the home because of care-taking responsibilities. For instance, one participant (W) argued that “Women should not be expected to stay home with their children. They can work and care for a child simultaneously.” The decision to stay at home or to work were often described as personal choices: “Women should not be restricted in their career or wish to work simply because they are held back by family life” (W). Another stated:

I disagree; women should not be required to stay home with their children; however, I did not pick strongly disagree because if a woman wishes to and that is the best decision for her family, then I have no issue. At the end of the day, it should be a family decision to decide if and who should stay home. (W)

As reflected by the above quotations, participants often did not believe that women should be required or restricted to staying at home. Several of the participants who felt that the mother should not be expected or required to stay home were women, while the few men who referenced that the woman should not be required to stay in the home supported their justification by noting that gender roles and/or stereotypes have changed and are less restrictive including for men, who are also potential caretakers. They also noted that there are opportunities to access outside help. For example, a few of the men noted,

I think that in today's world, there are a lot less "should’s" when it comes to gender roles. I don't think that the mother of a family is expected to be the one to stay home and watch the children anymore. (M)

I feel that a mother should be able to work wherever she wants and should not be limited to being a stay-at-home mom once she has a child. I also feel like the father, or a babysitter could come and take care of the child when he/she is not in preschool and the parents are still at work. (M)

### Mothers Should Work as Working Fathers Do

A portion of the participants believed that mothers can both work and have children and be successful in doing both. Participants asserted that having a career while raising children is not only possible but should be encouraged. Several women advocated for having both a successful professional life and a family life. For example, one participant wrote that, “They should have the ability to go out there and pursue their passions as well. There's nothing wrong with doing that and caring for your children. It is also possible to do so” (W). Another wrote, “I think that woman should be able to have a career and a home-life, a work life balance is very attainable” (W).

However, the belief that mothers should be able to work often co-occurred with the stated expectation that women should have the same options as men, where men are being used as the standard for what women should aim for. In other words, driving the expectation that mothers should be able to do it all was the assumption that they should work, just as working fathers do. However, many of their responses did not mention how the work at home would be managed if women work as men do. For example, one participant (W) stated that, “I disagree because I think women can work outside of the home and still have children. Many parents do it and there is no reason that women can do it just as men do.” Women expected mothers to be able to be like men, and to not be limited: “I believe that if a woman wants to have a successful career outside of the home like the man, then she should be able to. She shouldn't be limited to staying in the home” (W). Another suggested,

I think women have the right to pursue a career and a family at the same time and should be held to the same standard of going back to work as men. If she wants to stay home then great, but if she doesn't that's great too. (W)

The assumption that mothers should work as men do also often framed children and the need for childcare as a problem that was limiting or restricting to women. For example, one female participant stated that, “Having a young child should not prevent the mother to work outside. Mothers are not obligated to provide child care.” However, who should take on childcare was not specified. Similarly, another participant noted that having children should not limit women’s freedom:

I think women should not have to bear the brunt of having children. They should be able to go back to work, and not limit their work because they decided to have a child, when their spouse has the 'freedom' to go about their lives and not restrict their working

conditions. (W)

Participant responses revealed that women were making direct comparisons to men and used the opportunities that working fathers have as the standard for their expectations for work life.

### Where are the Fathers? Childcare is Mom’s (Not Dad’s) Problem

As suggested above, when considering a mother’s involvement in the workplace, participants perceived an underlying problem– children. Several participants felt that children should not inhibit a woman’s ability to work. For example, one participant stated that “Married women with preschool aged children should be able to work from home or wherever they like no matter the age of their child” (M). Another said,

I believe that if a woman wants to work AND be a mother she should be able to do both. Just because a woman has a child should not restrict them to staying home with them. Plenty of children, myself included, grow up going to daycare and turn out just as fine as the rest. (W)

As this last participant’s response suggests, participants may have been more open to alternative sources of care because of their own experiences with childcare that goes beyond only maternal care. In discussion of alternative childcare options, participants mentioned several possibilities, including preschools, babysitters, and even grandparents as possible sources of childcare, indicating that both men and women see many potential resources for childcare. For example, participants noted:

Women, married or not, should be able to have their own career and children at the same time. Men should be held to the same expectations when having little children. Women are able to do whatever they want; however old their children are. (W)

With the help and support of family members and trusted caregivers (i.e. nannies), there is no reason that a woman can't work full or part time in a job that she enjoys while also being able to care for and interact with her kids. The notion that women must work from home or can't hold a job because they have to be a full-time caregiver is outdated and does not account for single mothers or career-driving women. (W)

However, although women were described as free to work as a result of these other forms of childcare support, participants assumed childcare was a problem that women would have/ or had the onus to solve on their own in order to be able to work. In the following response we can see how participants assumed that the mother is the one expected to arrange for childcare:

While the child is still young, there are alternatives to the mother to care for the child. *She can choose* to hire someone or send them to preschool, and they can work, or *she can* watch them without a job, *it is up to her.* (authors’ emphasis, M)

As noted in the responses above, several participants assumed that the problem of childcare could be resolved by outsourcing childcare responsibilities. For instance, one noted, “If both parents are working then they can hire a babysitter but if one of the parents is not working then they stay with the kid at home” (F). Therefore, underlying the expectation of a mother’s choice was the assumption that childcare would be outsourced in order to support their professional goals.

Others were supportive of a mother working if her childcare responsibilities were taken care of in approved ways. For example, one participant stated that, “I would say that I disagree because if the child is in preschool then they would be able to attend work during those hours. I think having a babysitter for an hour or two is OK” (M). Men often argued that the woman could work while the child is at school (or being cared for by another). In other words, a mother’s ability to work was tied to and expected to be responsive to her childcare responsibilities, and therefore she could work if childcare was taken care of.

However, whereas many participants referenced the various systems in place (i.e., nannies, babysitters, schools) that allowed women to work, very few participants noted that the father could also provide childcare support. This is particularly striking, considering that as mentioned in the above, mothers’ freedom to work was assumed to be the same as working fathers, but men in general, and especially fathers, were rarely mentioned when addressing childcare concerns.

Overall, most participants treated childcare as either a problem that mothers (not fathers) should solve by doing managing her work schedule around childcare needs, or a problem that could be avoided by the mother arranging to have others do the childcare (e.g., preschool, grandparents, babysitters). Additionally, very few participants argued that childcare should be a shared responsibility of the mother and father. Therefore, childcare was often framed as an impediment that women would have to face and address rather than as a responsibility that should or could be shared by fathers.

### Mothers Can’t Choose to Stay Home

Rather than viewing working outside the home as a choice for mothers, some participants asserted that it is not only men’s responsibility to provide for the family, and that women should work also. Therefore, work was seen not as a personal choice, but as based on family needs. For instance, one participant (W) explained, “Women will have to work, along with their husbands to keep the family moving forward. Taking care of kids is not the sole responsibility of the women, they, like their spouse, should be able to balance both home and work life.” Similarly, another participant (M) explained, “Because moms need to have an income and provide for their children and they should send them to school.” The language of need as a reason for mothers to work was also present in responses that could be implying that such an arrangement was not ideal, or at least was not always expected. For instance, a participant noted, “If the household has financial needs to be met, then the woman of the house *can* work, which may require the child to be in daycare or after school programming. May be a good option to socialize the child” (M, emphasis added).

### Mothers Should Stay Home

Although most of our participants expected mothers to be able or have to work, some participants thought women should stay at home. A few women felt that the women should stay home with preschool aged children, often referring to the wellbeing of the child to support their reasoning. For instance, one participant (W) stated, “I believe that woman with preschool aged kids should be home as much as possible in order to care for them and give their child as much support as they need while growing up.” Child development and mother–child relationships were often referenced to support women’s reasoning as to why the wife should stay home. One participant who agreed that the women should stay home stated that, “This age is very influential in the development of a child. I think involvement is very important” (W). Another participant said, “I think that age period for a child and mother is crucial in relations to building a strong relationship. Mothers should be able to see and be aware of the developments going on in their child's life” (W). Given that there was no mention of fathers providing care, these participants seemed to see mothers as having a special obligation to take care of young children. Moreover, participants assumed that staying home was the primary way in which care-giving would allow for the building of relationships, or contribute to children’s development.

Interestingly, a few of the women who believed that women should stay home also acknowledged that women can work too, indicating that they may be experiencing some conflict or a desire to do it all. For example, these participants mentioned both caretaking as an obligation and work as either an obligation or an option: “I do believe that a married woman should be taking care of her preschool child, but at the same time, she needs to be providing for her family if she has a full-time job” (W) and “I think you should care for your kids at that age since they are developing but I think you should have the freedom to work to” (W). Thus, despite endorsing the statement that women should stay home, these women also mentioned work as a legitimate “freedom” women should have.

A few of the emerging adult men who believed that women should be homemakers often referenced gender norms—that is, the way they perceive things are most often done by men and women, without further justification about the benefits of this arrangement. For example, one participant (M) argued that, “Because the men work and the women clean. Kids also need a structure.” Most men who agreed that a woman should stay home with preschool-aged children did not focus on the wellbeing and development of the child. Instead, they relied more on norms related to gender as common and therefore correct, as exemplified in this example:

I believe that it is more common for the woman to stay home with a child and have the husband be the bread winner of the family. It is not a matter of a woman not being capable to do jobs outside the house besides being a mom but it is more common for the man to work outside the home and the wife to watch the children. (M)

Similarly, one participant suggested that, “women tend to be better nurturers than males and is rather instinctual. I think that it would be the most logical way” (M). Thus, many of the men who suggested that women should stay home made efforts to suggest that their position was based on women’s perceived abilities to be better at childcare, and not a belief that they are incapable of work.

In summary, most participants believed that mothers should be able to choose to work. Participants noted the various systems outside of the immediate family in place that could be used for others to engage in childcare but they also thought childcare was a mother’s problem to be solved, rather than an equal responsibility. In answering our first research question, our analysis suggests that most emerging adults in our study wanted mothers to work and to figure out the problem of childcare. Assumptions of balancing the two needs tended to prioritize work, to emphasize personal choice, and to note the various systems outside of the immediate family in place that could be used for others to engage in childcare to help women have it all. Some participants assumed mothers should schedule their work around childcare (or the systems in place that are available for certain periods of time). A few suggested that women should not work for pay and instead should prioritize childcare over work. Across participants who both agreed and disagreed with the statement that women should not work when they have preschool aged children, very few participants mentioned the expectation that childcare should be a responsibility that the father or another partner would also shoulder. In other words, women’s work continued to be seen in relation to their ability to address childcare needs, without much consideration for how working fathers (or another partner) could or would balance both considerations.

### Analysis of the Husband Does Housework Beliefs

The majority of participants disagreed with the idea that a working husband should not help his wife around the house. Most participants (69.33%; 57.3% women) either strongly, moderately, or slightly disagreed, whereas 12.50% (59% women) neither agreed nor disagreed, and the remaining 18.18% (25% women) either somewhat, moderately, or strongly agreed. Overall, then, most participants believed that a working husband should help around the house. Analyses revealed four major themes associated with participants’ assumptions underlying their views and explanations for their position: 1) housework is also a working man’s job, 2) wives are working (at home or otherwise) too, 3) a working husband should rest, and 4) housework should be shared. Each theme is considered in more detail below. (See Table 2 for a summary of major themes and code frequency).

**Table 2:** Themes and Coding Analysis for Husband does Housework Beliefs

**Major Themes**					
*Codes*	Description	Cohen’s Kappa	Counts/ Percentages		Exemplary Quote
**Housework is Also a Working Man’s Job**					
*Husbands should always help*	Refers to the idea that a husband should always help with housework	1.00	*20 (11.4%)*		“The husband should always help the wife, no matter what sex.” W
*Husbands can work and help*	Refers to a husband’s work status, but also notes that he is still capable of helping with housework after	1.00	*32 (18.2%)*		“Even if he had a hard day, he should still do his part at home.” W
*Wives need help*	Refers to the wife needing help to complete housework	1.00	*7 (3.8%)*		“The wife still needs help.” W
*Wives can’t do everything*	Refers to the idea that the wife cannot do all the housework and childcare by herself	.74	*6 (3.4%)*		“I get a long days work sucks, but if a woman is working too she should not be expected to do everything by herself. The man should also help if they are both in the same boat. Even if she doesn't work, it is disrespectful to his wife if he does not help at least a little bit.” W
*Wives can expect help*	Refers to the wife having the right to expect her husband to help	1.00	*5 (2.8%)*		“The wife should be allowed to have some expectations and deserves help around the house.” W
*Husbands must do something*	Refers to the husband having the responsibility to help around the house in some manner	.79	*7 (3.8%)*		“The wife could have worked hard all day too. Just because he is a man doesn't mean he is exempt from responsibility.” W
**Wives are Working Too**					
*Both partners work*	Refers to both spouses working hard or having full days at home and or at work	.71	*24 (14.6%)*		“If both partners work, then household responsibilities may get pushed off sometimes, but should still be a team effort. If the wife does have a less demanding job, then she may step in and do more housework, versus her husband with a time demanding job.” W
*Work from home is still work*	Refers to the wife laboring in the home as work	1.00	*11 (6.3%)*		“Just because the husband is going out to work doesn't mean the mother isn't working just as hard at home all day.” W
**A Working Husband Should Rest**					
*Husbands need rest*	Refers to a husband being excused from housework due to needing to rest after work	1.00		*17 (9.7%)*	“If he comes home from a hard day's work, and she is a housewife, then she should understand to let him take some time for himself. He has spend the entire day providing for the family.” M
**Housework Should be Shared**					
*Shared responsibility*	Refers to the expectation that chores should be a shared responsibility	1.00	*20 (12.5%)*		“being married is a two person things and they should share shores together no matter what. The wife shouldn't expect to do everything just because the husband came tired from work. She could've came home tired as well and not want to do the chores.” W
*Equal responsibility*	Refers to the husband and wife having equal responsibility in the home	.83	*19 (10.8%)*		“A husband is equally responsible for what needs to be done at home as the wife.” W

### Housework is also a Working Man’s Job

Many participants stated that the woman is not the only one responsible for maintaining the home and that the husband should always help despite working outside the home. For example, one participant noted, “His job is not done there, he has to care for the household as well” (M). Likewise, many women also stated that the husband should not expect the end of his paid working hours to be the end of his working day. Examples of these responses include: “A husband should provide assistance around the house and should not expect the wife to do all of the household chores” (W) and “The husband signed up to both have a career and a home. He should always help” (W). Note that in both examples, participants echoed the wording of the question by using the word “help” or “providing assistance” rather than indicating that they should each do an equal share.

Some participants also noted that the wife can expect the husband to help, as she should not be expected to complete all the household duties herself, even when the husband has had a hard day. For example, a participant argued, “A wife can expect whatever she may want from her husband, whether it be after a "hard day of work" or not” (W). Moreover, for some, the expectation that the husband should do housework extended to situations in which the wife did not have a paying job. For example, one woman presented the following scenario of a stay-at-home mother married to a father working full-time:

In the case where the husband is the primary breadwinner in the family, and the wife is a stay-at-home mom, the husband should not assume that he can sit back for the rest of the night. The wife also had a full day of responsibilities with taking care of household duties and the children. If the wife needs help when the husband comes home, it is his duty to help. He should not expect to be served when he comes home. That would mean the woman had a full day of working without any breaks, but the man finished working at 5:00 pm. A marriage should share the work equally, regardless of whose paycheck is bigger. (W)

Several participants assumed that the spouses had a family, and that assumption affected the work at home that needed to be done. Some challenged the notion that men should not have any household obligations once they come home when there are children to take care of. Many were resistant to having the husband rest while the wife had to care for the children and the household. For instance:

I understand that working full time is long and very tiring, but that doesn't mean the wife should be doing all the work. I wouldn't expect my husband to come home and lay on the couch while I take care of the kids and cook dinner and clean up and bathe the kids and put them to bed, etc. I would expect him to watch the kids while I cook or vice versa. After the kids are asleep, then both parents can take a break. (W)

Just because you have a day job does not mean you can come home and relax. You also signed up to be a father and raise a family so when you come home from your job, that’s your next job is taking care of your family. (W)

Overall, most participants believed that a hard day at work should not excuse the husband from doing chores. For instance, one participant noted, “It should be expected that both help each other out on tougher days and that partners should be trying to not let work stress affect family responsibilities” (M). Therefore, participants believed that a husband’s contribution to housework and home life should not be directly affected by his work outside the home.

### Wives are Working Too

Several participants noted that husbands should engage in household labor in the evening because wives are engaging in labor during the day as well. However, significantly more women than men in the sample mentioned the possibility that both spouses’ work. For example, one participant noted, “The woman comes home from a hard day's work also. It shouldn't just be subject to one gender” (W). Likewise, another participant stated that, “The wife may work just as hard as the husband, or the wife may even work harder. I think they should both take care of the house- e.g., cook and clean up kids messes” (M). Therefore, if the wife was noted as working, both her and her husband were expected to participate in chores.

In addition to considering the possibility that the wife also worked outside of the home, some participants noted that unpaid labor during the day (e.g., childcare) and working from home are still work, and thus, chores should still be shared. For instance, one participant stated that*, “*They share the house, therefore they both need to put in work. The wife still could have had just as hard of a day as the husband's workday even if she works from home, is a stay-at-home mom, etc.” (W). However, only rarely did men in the sample assume that the couple had children and mention that the wife working from home as the caretaker of children is hard work. Therefore, what was considered work was often gendered.

On the other hand, participants sometimes suggested that a wife’s working status impacts the chores she should be responsible for, and if the wife does not engage in paid labor, she should be fully responsible for the chores: “If she is not working then all the chores should already be done” (M) and “If the woman didn’t work at all then she should be doing the household chores” (M). Another participant suggested that the wife should do more, though not necessary all the household chores, “Household work should be a part of everyone's shared duties, although I do think it is the wife's responsibility to do more if she is a stay-at-home wife” (W). Therefore, several participants tied what chores the husband could do to whether the wife had completed them or not based on her own availability.

### A Working Husband Should Rest

Although participants typically agreed that men should help in the home, several participants assumed that the wife would do most of the chores to help her husband rest after a hard day. For example, one participant stated that, “If the husband had a hard day's work, the wife should comfort and take care of him. Maybe give him a massage, but definitely make dinner and clean” (M). Interestingly, typically participants who believe that the husband needs rest following a hard day at work were women. For example, one participant wrote, “If the husband does need a break after work, then the wife should respect that and not try to push chores on him. But he should also equally share chores when he has the time to” (W). For this participant, a concern with equality is most relevant when the husband has time. Similarly, another participant noted, “The husbands need to be cut a break if they return from extremely stressful jobs. If they are not beat, then they can help” (W). Therefore, several participants assumed the husband’s needs (e.g., to rest) should be prioritized. Expectations for sharing household work depended on when it was convenient for the husband.

Others noted that the desire to rest should be respected, but that a husband could not rest all the time. Several participants noted that the wife should allow her husband to rest sometimes. One said, “If the husband comes home everyday from a "hard day" that is not acceptable. But here and there that can be allowed” (W). Another noted,

The husband should expect to help around the house when coming home, but there are some certain circumstances where the male needs some time to himself and the wife can be respectful of this. This should also then go both ways though. (W)

Only moderately disagree because if it's a really tough day every once in a while and you know he doesn't want to be bothered, just give him the night off. Other than the extreme, he needs to be responsible for getting his share done around the house. (M)

A few men who advocated for a husband’s right to rest referenced traditional gender roles. Specifically, some men mentioned the husband’s position as the primary breadwinner of the family, as presented in the following examples: “Keeping a house together is a shared responsibility but if he is the only one bringing in money it might be best to let him have some nights off” (M) and “If he comes home from a hard day's work, and she is a housewife, then she should understand to let him take some time for himself. He has spend the entire day providing for the family” (M). The implication seems to be that earning money is more difficult or important, and therefore requires more rest than unpaid work. In fact, many participants assumed that the wife would be responsible for the chores until her husband had the time to contribute. Sometimes participants referenced a concern for fairness to support their assumption of the husband needing rest. For instance, “If the wife was home all day while the husband was hard at work, it is only fair to give him a slight break in this situation” (W).

### Housework Should be Shared

Only some participants mentioned that chores should be a shared or the equal responsibility of both spouses. Participants asserted in the following examples that because the home belongs to both of them, both spouses are responsible to contribute in some way: “The wife may work just as hard as the husband, or the wife may even work harder. i think they should both take care of the house- e.g., cook and clean up kids messes” (M) and “The husband should at least try to do things around the house because it's a group effort, not just for the wife to do. Chores are because of the whole family and it is everyone's responsibility” (W).

However, gender differences emerged. Oftentimes, those who said that the chores should be shared or equal were women. For some, the expectation for equality in chore participation was based on the wife’s work status, as this justification suggests: “It depends on working situations: I believe stay at home mothers should take care of the majority of household chores if they are not working. If the mother does work, the father should help equally with housework” (W). For these participants, equality in chores is only necessary when both spouses’ have jobs, and even more, jobs that pay equally and/or are equally difficult. Another participant also noted that household responsibilities depend on who works, as well as the difficulty of each job and the pay earned:

This depends on the job of both the husband and the wife, but I do not think the wife should expect the husband to do this if he has the harder job that pays for the majority of expenses. I think the scale is more tipped to the wife doing most of the work if she does not have a job. However, it could be tipped the other way if they both have equal amounts of work in their jobs. Then, the chores would be split more evenly. (M)

In summary, many participants believed that a husband should help with household chores even if he worked full-time. The word “help” implies that the major responsibility belongs to someone else; however, very few challenged the notion that help was not enough, or suggested that the husband should do an equal amount. Moreover, participants often framed the husband’s involvement in relation to his wife, and particularly his wife’s work status. When participants advocated for more equal participation from the husband, it was often tied to the daily activities of the wife—particularly work for pay. In fact, the pay and difficulty of work by both spouses was often key to determining how much they should contribute to the household, and husbands who were the primary breadwinners were seen as especially deserving of rest. The exception to this seemed to be when participants assumed the couple had children. Participants who mentioned children often considered contributions (and sometimes equal contributions) from the husband to be required. Some noted that the chores should be shared or equally divided simply because the home is both of theirs, and therefore a shared responsibility. Therefore, in answering our second research question, the results suggest that emerging adults expect working men to help with housework, but also expect both partners to make allowances for the demands of the workplace, as well as childcare demands. In particular, men’s involvement in housework was relational—participants considered his involvement in the home to be based on not only his career (he can rest, he has to at least help after work), but his wife’s career as well.

## Discussion

The main purpose of this study was to investigate the underlying assumptions of U.S. heterosexual emerging adults attending a PWI institution about balancing the dual demands within the home and the workplace. The results indicated that overall, emerging adults believe that mothers of preschool-aged children should have the choice to work, and that working husbands should help their wives to do housework. These findings are consistent with recent gender attitudes in support of working women (Dernberger & Pepin, 2020) and the expectation that husbands should help with the chores even if they are working (Midgette, 2020; Wenhold & Harrison, 2020). However, thematic analysis of participants’ open-ended responses about the demands of work life and home life suggested that working mothers were expected to model men’s roles in the working world without the support from home that many men receive. In other words, they were expected to not let children “limit them,” whereas very few participants noted the possibility or expectation of a second partner to support childcare needs, and only a few mentioned that “helping” is not the same as contributing equally. Moreover, many participants considered work contributions as a legitimate reason for avoiding housework. Overall, in both situations, participants tended to expect flexibility from the family, and especially the wife, in response to work-life requirements.

Few participants relied explicitly on gender stereotypes when explaining how family life and work life should be balanced. However, emerging adults made sense of how life should be balanced in gendered ways, as suggested by the prior literature: working husbands should be helpers (Midgette, 2020; Wenhold & Harrison, 2020), but not expected to stay at home to do childcare (Fulcher & Coyle, 2011; Fulcher et al., 2015), whereas mothers can be workers (Gere & Helwig, 2012), if not limited by children (Ezzedeen et al., 2018). Together, these findings suggest the development of a one-sided form of egalitarianism that shows preference and valuing of career and autonomy of men and women to some degree (i.e., optional or not to be limited), rather than a balancing of both career needs as well as family members’ care needs.

Although attitudes among younger generations have been found to be supportive of working mothers (Scarborough et al., 2021), the contemporary views of participants in this study suggest that little progress has been made regarding how men will contribute to women’s work-life balance. This is evident in the current absence of men in communal roles (Croft et al., 2015) and the unequal division of domestic labor in American dual earner households where women are still performing more household duties than men, including more laundry, cooking, cleaning, and washing the dishes (Brenan, 2021). Although in dual-earner households where spouses have similar incomes, men are more likely to help with household chores (Brenan, 2021), the language of “help” is problematic. “Helping” perpetuates these gender inequalities in the home because it maintains that the chores are primarily the women’s job (Miller & Carlson, 2016) and lets men off the hook as far as being equally responsible for the household. In the current study, few participants questioned the idea of men “helping” as the primary form of engagement in chores, thus supporting previous findings that emerging adult women expect to take the primary responsibility for chores in their future relationships (Askari et al., 2010; Dernberger & Pepin, 2020; Fetterolf & Eagly, 2011).

As argued by recent scholarship, much of the focus on gender parity has concentrated on women engaging in the workforce, and less on men’s engagement in communal and family roles (Croft et al., 2015; Meeussen et al., 2020), such as those of childcare. Our findings indicate that at the individual level, emerging adults also treat gender parity as a woman-problem, rather than a relational one (i.e., to be worked out between the spouses with both contributing substantially at home). We argue that the language of women’s *choice* to work contributes to the notion that work is optional for women, and it only becomes an option if they organize the family’s childcare. Many of participants’ responses did not consider the obligations of fathers to play a substantial role in their children’s care. Thus, the participants rarely considered men’s role in contributing to women’s work-force engagement. Together, these assumptions help explain how despite endorsing egalitarian attitudes, college-attending heterosexual emerging adults in a U.S. PWI continue to expect gendered roles when balancing family and work life with the burden of this balancing act placed on the women.

### Contextualizing Heterosexual Relational Assumptions

As pointed out by several participants in this study, the gender relations assumed within participants’ responses were also often classed and raced. For example, the language of “choice” to work was questioned by several participants, who noted that not all women had the financial choice to not work. In fact, recent research has noted class differences in expectations for engaging in household labor (Miller & Carlson, 2016), highlighting that class may influence both men and women’s gender ideology about housework. For instance, most of our participants agreed that men should help with chores, which has been found to be more likely to be the case in families where men attended college than in those in which they have not (Miller & Carlson, 2016). Moreover, prior research has shown racial and ethnic differences in how American families divide household labor: African American families tend to have the lowest housework gap between heterosexual couples, followed by European American, Asian American, and Latinx couples (Wight et al., 2013). Studies have suggested that these racial-ethnic differences may be in part explained by cultural differences in attitudes about the value of housework (Perry-Jenkins et al., 2013), early messages socializing boys when they were younger, and expectations for early engagement in household involvement and competence (Penha-Lopes, 2006). Therefore, it is likely that in our primarily White sample, students’ attitudes may be influenced by their prior cultural and ethnic socialization, and in another sample other assumptions may be underlying expectations for balancing work and family life.

The current study contributes to the literature on the outsourcing of family labor by demonstrating how the next generation of college-attending primarily White heterosexual men and women in our sample assumed that women should continue to attend to the issue of childcare as a personal problem by outsourcing the labor to others. As reflected in prior research, outsourcing childcare has become a strategy for balancing work and care for dual- earner couples (Harbach, 2012; Raz‐Yurovich, 2014), and provides options for families with personal, economic, or structural (i.e., work) barriers or based on their personal preferences (Harbach, 2012). Studies have shown that public preschool increases mothers labor force participation and decreases female career sacrifices (Malik, 2018), while full-day kindergarten makes it more likely for women to work full-time when compared to half-day programs (Cannon et al., 2006). Participants in this study acknowledged the complexity and heterogeneity of familial situations that contribute to childcare decision making as emphasized in past literature (Harbach, 2012). Additionally, participants expressed that there is more than one way of caring for a child, citing preschools, daycares, babysitters, and other family members as means of providing care (Harbach, 2012), yet rarely suggested the father provide child care, reflecting the trends of families using babysitters and other family members (i.e., grandparents) over fathers to care for their children when the woman is working (Laughlin, 2010). Thus, our sample supported the outsourcing of childcare to make it possible for mothers to work, but they did not assume childcare needs would be shared with or met by fathers.

### Limitations and Future Research Directions

One of the main contributions of this study was the use of open-ended responses that allowed us to gain insight into U.S. heterosexual college-attending emerging adults’ underlying assumptions about balancing work and family life within heterosexual households. However, this study had limitations. As this study relied on short, open-ended questions that were part of a longer survey, future research should include longer in-depth interviews to gain greater understanding of how emerging adults are making sense of their future work-life balance and household dynamics. In addition, each situation we provided to participants focused on one gender, and it would be interesting to investigate how emerging adults would respond to less traditional situations such as a wife who wants to relax after a hard day or a husband’s expectations about work outside the home when he has preschool aged children. Future research should further explore what contributes to expecting family-life to be responsive to work-life, rather than work-life being responsive to family-life, and whether the expectations of which realm should be most responsive differs when considering husbands versus wives. Moreover, future research should consider items and questions that would include egalitarian situations as well as diverse family formations (e.g., single-parent family, same-sex couples, multi-generation family).

Additionally, this study was limited to a specific sample at a single point in time. To better understand how emerging adults’ gender ideologies contribute to expectations and actual future divisions, future longitudinal research is needed. This study was also limited to primarily White heterosexual emerging adult men and women attending a PWI in the United States. As evidenced by a recent review of work and family research in the second decade of the twenty-first century (Perry-Jenkins & Gerstel, 2020), more research is needed to investigate race and class heterogeneity. In addition, prior research suggests that same-sex families tend to be more egalitarian in their division of household tasks than different-sex families (Goldberg et al., 2012; Perlesz et al., 2010). Investigating sexual minority emerging adults’ understanding of work and family balance may provide additional insight into how families can balance the two forms of labor and create more egalitarian divisions. Finally, future research is needed to investigate what factors contribute to considering childcare as a responsibility to be worked out by all parents where parity can also be expected. In line with Fulcher et al. (2015) suggestion that the cultural expectation of gendered roles in the home has not advanced as far as the roles in the workplace, our work underscores the need to further investigate what contributes to cultural expectations for men’s involvement in family life.

### Practice Implications

The expectation that childcare is a problem that women are responsible for solving aligns with prior findings that the gendered division of labor in the home, specifically care work, is both expected and accepted by the public (Croft et al., 2015), and is evident today during the COVID-19 pandemic as mothers are often the ones to stop working in order to care for their children (Collins et al., 2020). Our findings suggest that the burden will continue to fall on women to “choose” to take on family responsibilities as long as several assumptions remain unchallenged. Rather than aiming to encourage families and the next generation of emerging adults to support women’s involvement in the workplace, the assumption that women should be able to “choose” to work or care for their children should be questioned. Both policymakers and educators should consider practices that support and encourage the role of men in addressing childcare needs, so that work and home life can go beyond a woman’s choice (or problem). For example, workforce policies such as lack of access to paid paternity leave for fathers would require men to stay at work and perpetuates the male breadwinner norm (Bartel et al., 2016).

Similarly, there is a need to challenge the assumption that men should “help.” As many scholars have noted (e.g., Midgette, 2020; Miller & Carlson, 2016), referring to a husband’s contributions as “help” was reflected in our data and contributes to the continued gendering of household labor. Moreover, in discussing career development and future plans, high school educators and career counselors should consider moving away from focusing on only career expectations, and also discussing family involvement considerations; as thinking and planning for family involvement has been suggested to potentially explain college-attending men’s greater involvement in household labor (Miller & Carlson, 2016). Finally, as noted by many feminist scholars, personal choices are in fact political, and how the family is run influences the larger systems in place (Bianchi et al., 2012; Okin, 1989). Social justice courses, courses on ethics, and others should consider discussing how choices within the family, such as helping and expecting flexibility in heterosexual households are linked with the gendering of labor within and outside of the family, and ultimately contribute to society's current class, race, and gender relations (Coltrane, 2000).

### Conclusion

Overall, our study found that US heterosexual emerging adults attending a PWI expected childcare to be a problem for a working mother to solve (or to outsource to others to solve), and they rarely mentioned the role of the working fathers in childcare to support the family and their working spouse. Overall, our findings suggest that the husband’s involvement in family life was tied to his wife’s involvement but did not often state the same expectations for a wife’s involvement in either work or family life to be tied to her husband’s involvement. Therefore, when considering how families come to balance paid and unpaid labor within and outside the home, as recommended by Croft et al. (2015), we need to move beyond emphasizing women’s roles and choices to consider the role of men in more communal roles in the home. Ultimately, our study suggests that addressing potentially unspoken assumptions about who should and can care within the family can serve as a potential catalyst for accelerating the gender revolution.

## Data Availability

The authors can be contacted to share the codebook and materials.
